# Interdependence of Contributing Factors Governing Dead-End Fouling of Nanofiltration Membranes

**DOI:** 10.3390/membranes11010047

**Published:** 2021-01-12

**Authors:** Oranso Themba Mahlangu, Bhekie Brilliance Mamba

**Affiliations:** Florida Science Campus, Institute for Nanotechnology and Water Sustainability, College of Engineering, Science and Technology, University of South Africa, 1709 Roodepoort, South Africa; orathem@gmail.com

**Keywords:** dead-end filtration, cake-enhanced concentration polarization, affinity interactions, synergistic effects

## Abstract

Cake-enhanced concentration polarization (CECP) has been ascribed as the main cause of flux decline in dead-end filtration. An unfamiliar approach was used to investigate the role of CECP effects in the fouling of a nanofiltration membrane (NF-270) that poorly reject salts. Membrane–foulant affinity interaction energies were calculated from measured contact angles of foulants and membrane coupons based on the van der Waals/acid–base approach, and linked to resistance due to adsorption (*R_a_*). In addition, other fouling mechanisms and resistance parameters were investigated using model organic and colloidal foulants. After selection, the foulants and membranes were characterized for various properties, and fouling experiments were conducted under controlled conditions. The fouled membranes were further characterized to gain more understanding of the fouling layer properties and flux decline mechanisms. Sodium alginate and latex greatly reduced membrane permeate flux as the flux declined by 86% and 59%, respectively, while there was minor flux decline when aluminum oxide was used as model foulant (<15% flux decline). More flux decline was noted when fouling was conducted with a combination of organic and colloidal foulants. Contrary to other studies, the addition of calcium did not seem to influence individual and combined fouling trends. Foulants adsorbed more on the membrane surface as the membrane–foulant affinity interactions became more attractive and pore blocking by the foulants was not important for these experiments. Hydraulic resistance due to cake formation (*R_c_*) had a higher contributing effect on flux decline, while CECP effects were not substantial.

## 1. Introduction

Membrane technology, especially nanofiltration (NF) and reverse osmosis (RO), have become promising advancements in water treatment. These processes remove most organics from contaminated water [1]. Two filtration modes are common in membrane filtration, namely cross-flow filtration and dead-end filtration modes. According to Blankert, dead-end filtration is a hopeful technology that can be applied as a conventional water treatment process [2]. However, dead-end filtration is more susceptible to fouling compared to the cross-flow filtration mode and this limits its application. Fouling of nanofiltration membranes in cross-flow filtration has been widely investigated [3,4,5]; however, there are few studies focusing on dead-end fouling of nanofiltration membranes. Membrane fouling has been categorized into external surface fouling and pore blocking, where complete pore blocking, incomplete pore blocking, and standard pore blocking are the major pore blocking mechanisms responsible for flux decline [6].

Organics and colloids are the major foulants dominating fouling of NF/RO membranes. These foulants accumulate on the membrane and change the membrane surface properties, thereby resulting in reduced permeate flux, poor solute rejection, and reduced membrane life span. In most instances, researchers have preferred using foulants mimicking polysaccharides due to their abundance in natural waters, and sodium alginate has been used as a model polysaccharide. Sodium alginate is composed of 61% mannuronic acid and 39% guluronic acid, and has a molecular weight distribution between 12 and 80 kDa [7]. The structure of sodium alginate changes to a highly organized gel in the presence of calcium ions [8], through the preferential binding of calcium ions to the carboxylic functional groups of alginate. Some researchers have reported this behavior to aggravate fouling [4,9,10], while other researchers believe that this complexation effect minimizes the impact of fouling on flux decline due to the formation of larger alginate complexes with low resistance to water passage [1,11,12]. Carboxylated styrene butadiene (CSB) latex may foul the membrane due to its physico-chemical properties. Latex particles have smooth spherical shapes. These particles are stable due to electrostatic forces [13]. Aluminum oxide is another type of colloids that may potentially foul membranes when present in the feed solution. These colloids consist mainly of α- Al_2_O_3_ with a surface area of 85 to 115 m^2^ g^−1^, iso-electric point between pH 8 and 9, and density of 3.2 g cm^−3^ [14].

Though membrane fouling has been widely investigated for organic and colloidal foulants in cross-flow filtration [4,5,9,10,15,16,17], fouling mechanisms (especially in dead-end filtration) are not yet fully explained due to complexities and several factors that play roles in membrane fouling [18]. It is for this reason that fouling is investigated in dead-end filtration mode in order to verify whether fouling mechanisms in the dead-end filtration mode are similar to those in the cross-flow filtration mode. Listiarini and co-workers investigated fouling mechanisms and resistance analysis in dead-end filtration of NF membranes using sodium alginate, calcium, alum, and their combinations. Flux decline was attributed to cake formation with compression and pore blocking [1]. Asatekin and co-workers carried out dead-end fouling experiments of NF membranes synthesized from polyacrylonitrile-graft-poly(ethylene oxide). Bovine serum albumin (1000 mg L^−1^) was used as model foulant and its fouling propensity on the membrane was not remarkable. However, a huge decline in flux was observed when Sepro PAN-400 membrane was fouled with the same foulant, indicating that fouling propensity also depends on the membrane properties [19]. Li and Elimelech (2004) also carried out natural organic matter (NOM) fouling in dead-end filtration. The authors found that the presence of divalent cations as well as the ionic composition of the feed solution controlled membrane fouling [15].

In this study, contributing factors leading to flux decline in dead-end fouling of nanofiltration membranes were further investigated. Combined fouling became the main priority as this subject has not been widely addressed in previous research work, especially in dead-end filtration. The main interest was to gain more knowledge on the fouling propensity of organic and colloidal foulants as well as their combination on fouling of nanofiltration membranes, and possibly understand the major contributing factors leading to flux decline. In depth characterization of the cake layers was undertaken to isolate specific cake parameters promoting flux decline and how these parameters are interdependent. Cake parameters as well as flux decline profiles were related to foulant properties as well as membrane properties. In addition, the evidence and the effects of cake-enhanced concentration polarization on membrane fouling were predicted by comparing NaCl rejection for the virgin and fouled membranes at similar fluxes. This unique approach has not been reported in dead-end fouling of NF/RO membranes. Finally, major conclusions and recommendations were made based on fouling and characterization results.

## 2. Materials and Methods

### 2.1. Foulants: Selection and Characterization

Sodium alginate (Sigma–Aldrich, Johannesburg, South Africa), polystyrene carboxylated latex (EOV group, Industrial park, B9700, Oudenaarde, Belgium), and aluminum oxide (Evonik Degussa GmbH, Hanau-Wolfgang, Germany) were selected as model foulants for the fouling studies.

Prior to conducting fouling experiments, it became fundamental to identify the critical coagulation concentration (CCC) of latex to avoid the modification of the membrane surface by excess calcium ions. Specific volumes of calcium chloride solutions (Sigma–Aldrich, Johannesburg, South Africa) were added to 20-mL sample vials containing 400 mg L^−1^ latex solutions to make final concentrations of 0, 0.001, 0.006, 0.011, 0.055, 0.111, 0.555, 11.098, and 110.98 mg L^−1^ CaCl_2_. The turbidity of the solutions after the addition of calcium chloride was measured (Eutech TN-100 turbidimeter, Thermo Fisher Scientific, Karlsruhe, Germany) and plotted against coagulant concentration to determine the critical coagulation concentration.

The electrophoretic mobility (*U_E_*) of the foulants (266 alginate and 400 mg L^−1^ for both latex and Al_2_O_3_) was determined at various pH values utilizing a Zetasizer 2C (Malvern Panalytical, Malvern, United Kingdom). The desired pH was obtained by addition of either ACS grade HCl or NaOH (Sigma–Aldrich, Johannesburg, South Africa). The Helmholtz–Smoluchowski Equation (1) was used to relate the (µm s^−1^ cm V^−1^) of the foulants to the particle zeta potentials [20].

(1) $$U_{E}=\frac{2\zeta \varepsilon f\left(ka\right)}{3\mu }$$

where *ζ* is the zeta potential (mV), *f*(*ka*) is the Henry’s function, *ε* is the permittivity of water (C^2^·N^−1^·m^−2^) (defined as *ε* = *ε*_0_·D, where ε_0_ is permittivity of vacuum = 8.85 × 10^−12^ (C^2^·N^−1^·m^−2^), and D the dielectric constant of water = 78.55 at 25 °C), and µ is the electrolyte viscosity (Pa.s).

Foulant sizes were measured by photon correlation spectroscopy (PCS 100 M, Zetasizer 2C, Malvern Instruments, Worcestershire, UK) at a foulant concentration of 200 mg L^−1^ and pH 6.8. The Zetasizer 2C has a resolution between 0.05 μm and 3.5 mm. Particle size measurement was based on laser light scattering where particles in a light beam scatter light into space, with scattering angles and intensities dependent on the particle size, the optical properties of the particles, the light source, and the medium in which the foulants are suspended.

The individual foulants (1 g L^−1^) were deposited on the NF-270 membrane coupons by filtering a 200-mL solution in a dead-end filtration system. The membranes with deposited foulants were dried in a desiccator overnight before contact angle measurements. Contact angle measurement was based on the sessile drop method where a minimum of 10 drops per liquid (water, glycerol, and diiodomethane) were placed on the filtered lawns using a microliter syringe (DSA 10-MK2, Kruss, Germany). The drop volume was 10 μL for all liquids and the measurements were conducted at room temperature. An average (±standard deviation) from the measured contact angles was calculated.

### 2.2. Membrane Selection and Characterization

A commercially available NF-270 membrane (NF-270, Dow Filmtec, Minneapolis, MN, USA) was selected to represent nanofiltration membranes. Information provided by the manufacturer revealed that the membrane is a polyamide thin film composite nanofiltration membrane that rejects 97% of MgSO_4_ under the following test conditions: 2000 mg L^−1^ MgSO_4_, applied pressure of 480 kPa, temperature of 25 °C, and 15% recovery. According to Nghiem and co-workers, the membrane has a molecular weight cutoff of 200–300 g mol^−1^, average pore diameter of 0.84 nm, and surface roughness of 4.1 nm [21].

To determine the membrane pure water flux (*J*_0_, Lm^−2^h^−1^), membrane resistance (*R_m_*, m^−1^), and NaCl rejection (%), the membrane was first compacted with deionized water at 500 kPa until stable flux. Next, the pure water flux was determined using deionized water at varying applied pressures and *J*_0_ was computed from the volume of water collected (*V*, L) at a specific time (*t*, s) and predetermined membrane area (*A*, m^2^) according to Equation (2). Equation (3) was used to estimate the membrane resistance (*R_m_*) with ∆*P* and *µ* being the applied pressure (Pa) and viscosity of water (Pa.s), respectively [22]:

(2) $$J_{0}=\frac{V}{At}$$

(3) $$J_{0}=\frac{\Delta P}{\mu R_{m}}$$

The rejection of NaCl was measured by filtering a solution of 2000 mg L^−1^ NaCl through the NF-270 membrane and Equation (4) was used to estimate rejection for the clean membrane (*R*_0_) from the measured electrical conductivity (µS cm^−1^) of the feed (*C_f_*) and permeate (*C_p_*). Equation (4) was not corrected for concentration polarization (CP) effects, which were less substantial for virgin membranes. A Consort C6010 conductivity meter (Consort, Turnhout, Belgium) was used to measure electrical conductivity.

(4) $$R_{0}=\left(1-\;\frac{C_{p}}{C_{f}}\right)\times \;100$$

**Figure 1:**
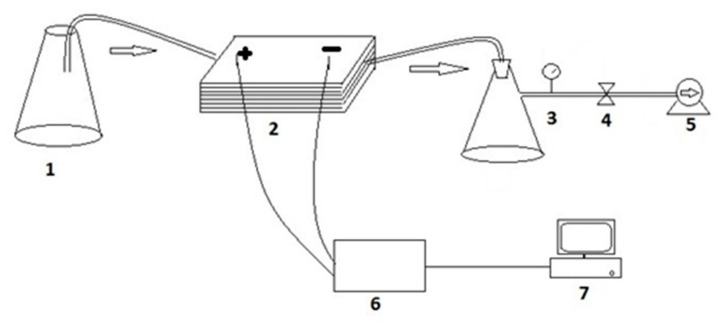
Self-assembled streaming potential: 1—Conical flask; 2—streaming potential cell; 3—pressure gauge; 4—valve; 5—vacuum pump; 6—analog digital converter; 7—computer.

The membrane zeta potential was determined before fouling at different pH values. The pH of the background electrolyte was adjusted using ACS grade HCl or NaOH (Sigma–Aldrich, Johannesburg, South Africa). The zeta potential was determined by the measurement of streaming potential (the potential at the shear plane between the membrane and the solution) using a self-assembled streaming potential analyzer (Figure 1) at a background electrolyte of 10 mM KCl (Sigma–Aldrich, Johannesburg, South Africa). Zeta potential measurements were done at room temperature. The measuring unit was equipped with a 6B11 analog digital converter (Analog Devices GmbH, Germany). The converter had an accuracy of 0.01 mV. The streaming potential cell had the following dimensions: Channel length of 6.42 × 10^−2^ m, channel width of 2.54 × 10^−2^ m, and channel height of 5 × 10^−4^ m. The tangential mode of analysis was used at a pressure range of −1 to −20 kPa. Briefly, the test solution was allowed to flow through the membrane surface by opening the valve. During the flow mode, a streaming potential (mV) was recorded in the data logger. A second measurement was made in non-flow mode (i.e., when the valve was closed). The difference between the potential in flow and non-flow modes was used in the calculation of the membrane zeta potential (ζ, mV) based on the Helmholtz–Smoluchowski Equation (5) [20]:

(5) $$\zeta =\;\frac{\Delta E\cdot \mu \cdot \delta }{\Delta P\varepsilon }$$

where ∆*E* is the measured streaming potential (mV) and *δ* is electrical conductivity (µS cm^−1^).

Membrane coupons (virgin and fouled) were characterized for hydrophilicity by measuring the contact angles at room temperature using a goniometer (DSA 10-MK2, Kruss, Germany). Contact angles were measured using the sessile drop method following similar procedures as explained in Section 2.1. Surface tension components and interfacial free energies of interaction between the membrane and foulants were calculated using the Lifshitz–van der Waals/acid–base approach as explained in our previously published work [4] and other studies [16,23].

Fourier transform infrared spectroscopy (Perkin Elmer, FT-IR 100, PerkinElmer Inc., Shelton, CT, USA) was utilized to record IR spectra of membranes in the wavenumber range 800–4000 cm^−1^ at 25 °C in order to elucidate changes in chemical composition of the membrane as a result of fouling.

Images of the membranes (virgin and fouled) were recorded using a scanning electron microscope (VEGA3 TESCAN, TESCAN, Orsay, France) with an irradiation beam of 20 kV. Prior to analysis, the membrane samples were dried in a desiccator for 24 h and carbon-coated to make them conductive. The quality of dispersion and the presence of foulants on the membrane surface were evaluated by energy dispersion of X-ray (EDX) (VEGA3 TESCAN, TESCAN, Orsay, France). To obtain a comprehensive elemental composition, EDX analysis were performed at different locations of the membrane surface.

### 2.3. Fouling Experiments

#### 2.3.1. Filtration Protocol

**Figure 2:**
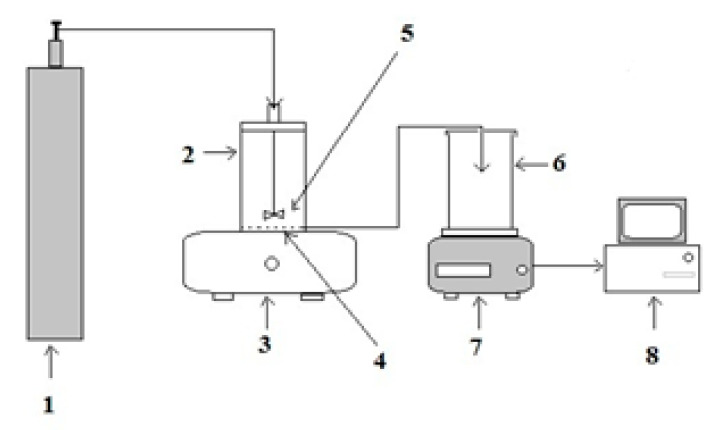
Schematic diagram of the dead-end nanofiltration setup: 1—Nitrogen gas cylinder; 2—dead end cell; 3—stirrer; 4—membrane sheet; 5—magnetic stirrer; 6—permeate collection vessel; 7—weighing balance; 8—data logger.

Nanofiltration experiments were carried out in a batch cell with a capacity of 300 mL (Sterlitech, Sterlitech Corporation, Kent, Washington, DC, USA) using NF-270 membranes. The disc membrane had a diameter of 320 mm with an area of 8.04 × 10^−4^ m^2^. The experiments were carried out under a fully conditioned environment to maintain a constant temperature of 25 °C. The applied pressure was monitored at 300 kPa using pressurized N_2_ gas. The dead-end experimental filtration setup is shown in Figure 2. In all the experiments, the feed was stirred to minimize the effects of concentration polarization on flux decline and salt rejection.

Fouling was performed with the selected foulants and NaCl (Sigma–Aldrich, Johannesburg, South Africa) was used as a background electrolyte at 10-mM concentration. In conducting fouling experiments, 200 mL of the respective fouling solutions was filtered through the membrane. To ensure that all the foulants were deposited on the membrane surface, a 300-mL graduated beaker was used to collect permeate. The experiments were stopped when the permeate level reached the 200-mL mark or when the weighing balance (Ohaus AX224, Ohaus, Columbia, MD, USA) read 200 mg. Fouling experiments were repeated in the presence of 0.5 mM CaCl_2_ (Sigma–Aldrich, Johannesburg, South Africa), except for fouling with Al_2_O_3_. Combined fouling experiments were performed with organic and colloidal foulants in the presence and absence of calcium (Table 1).

The pure water flux of the membrane before and after fouling was compared to investigate the effect and extent of fouling on membrane properties. The flux for the fouled membranes was estimated from Equation (2) by recording the time taken to collect a specific volume of permeate (deionized water) filtered through the fouled membranes at applied pressure of 300 kPa. The fouling index (*FI*) was then determined based on Equation (6) [24].

(6) $$FI=\frac{J^{\prime }}{J_{0}}$$

where *FI* is the fouling index and *J*_0_ and *J′* are the pure water fluxes of the clean and fouled membranes, respectively.

#### 2.3.2. Investigating the Influence of Cake-Enhanced Concentration Polarization (CECP)

The role of cake-enhanced concentration polarization was investigated by comparing NaCl rejection by the virgin membrane and the fouled membranes at similar fluxes. NaCl was chosen because the salt is poorly rejected by the NF-270 membrane. The aim was to investigate if the effects of CECP are important on fouling of membranes that poorly reject salts. This is because most researchers often cite CECP effects as the major contributor to both declines in permeate flux and salt rejection. Briefly, after membrane compaction as described in Section 2.2, the filtration unit was filled with 200 mL of 10 mM NaCl and rejection experiments were conducted at different permeate fluxes (which were obtained by adjusting the applied pressure). For the fouled membranes, NaCl rejection was investigated by carefully pouring 200 mL of 10 mM NaCl into the cell with the fouled membrane (ensuring that the fouling layer was not disturbed). This was followed by collecting 10 mL of permeate at 300 kPa applied pressure, and rejection was estimated from the measured electrical conductivity of the permeate and feed (Consort, Turnhout, Belgium) according to Equation (4).

### 2.4. Resistance Analysis

Membrane hydraulic resistance (*R_m_*, m^−1^), resistance due to adsorption (*R_a_*, m^−1^), resistance due to pore blocking (*R_p_*, m^−1^), and resistance due to cake formation (*R_c_*, m^−1^) are related to the trans membrane pressure (∆*P*, Pa), dynamic viscosity (*µ*, Pa.s), and permeate flux of the fouled membrane (*J′*, Lm^2^-h^−1^) based on Equation (7) [1], where *J’* denotes permeate flux for the fouled membranes.

(7) $$J^{\prime }=\frac{\Delta P}{\mu \left(R_{m}+R_{a}+R_{p}+R_{c}\right)}$$

Resistance determination was carried out in a series of steps as follows: Step 1: Determination of *R_m_*; step 2: Determination of *R_a_*; step 3: Determination of total resistance (*R_t_*); step 4: Determination of *R_p_*; and step 5: Determination of *R_c_* as explained in the next sections. The viscosity of the feed solutions was measured using a Brookfield Digital Viscometer, DV-II (Brookfield Engineering Laboratories, Stoughton, MA, USA).

#### 2.4.1. Determination of Resistance Due to Adsorption (*R_a_*)

After compaction as described in Section 2.2, the membrane resistance (*R_m_*) was determined from Equation (3) by measuring the water flux at 300 kPa. This was followed by analysis of resistance due to adsorption (*R_a_*) which was estimated as follows: 200 mL of the respective model foulant solution (Table 1) was poured into the filtration cell and stirred for 24 h at 200 rpm and 0 Pa. The foulant solution was then decanted and replaced with 200 mL deionized water and the permeate flux after foulant adsorption was determined at 300 kPa. The resistance due to adsorption (*R_a_*) was calculated based on Equation (8):

(8) $$R_{a}=\;\frac{\Delta P}{\mu J^{\prime }}-R_{m}$$

#### 2.4.2. Determination of Total Resistance (*R_t_*)

After determining *R_a_*, 200 mL of foulant solution was filtered at 300 kPa through the membrane coupon previously used in the determination of *R_m_* and *R_a_*. The permeate volume was measured to ensure that all the water was filtered through and the foulant particles were deposited on the membrane surface. After the fouling experiment, deionized water was poured into the filtration cell, taking care not to disturb the fouling layer and the pure water flux after cake formation was determined at 300 kPa. The pure water flux was based on the total resistance which constitutes of *R_m_*, *R_a_*, *R_c_*, and *R_p_*.

(9) $$R_{t}=\frac{\Delta P}{\mu J^{\prime }}$$

#### 2.4.3. Determination of Resistance Due to Pore Blocking (*R_p_*)

To determine the resistance due to pore blocking, the membrane coupon used in the previous steps was gently wiped with Kimwipes to remove the foulant layer on the membrane surface and rinsed with deionized water several times until no foulant was visible on the membrane surface. This was followed by determination of membrane pure water flux after pore blocking with deionized water at 300 kPa and *R_p_* was estimated from Equation (10).

(10) $$R_{p}=\frac{\Delta P}{\mu J^{\prime }}-R_{m}-R_{a}$$

#### 2.4.4. Determination of Cake Resistance (*R_c_*)

Cake resistance was calculated based on Equation (11), with *R_m_*, *R_a_*, *R_p_*, and *R_t_* having been determined in the previous steps [1].

(11) $$R_{c}=R_{t}-R_{m}-R_{a}-R_{p}$$

The fouled membrane coupons were characterized for SEM, EDX, and FTIR as explained in Section 2.2. Further characterization was done for selected cake parameters such as the specific cake resistance and mass of foulant deposited on the membrane surface. The specific cake resistance (α, mkg^−1^) was calculated based on Equation (12) [25].

(12) $$\frac{t}{V}=\frac{\mu R_{m}}{\Delta PA}+\left[\frac{\mu \alpha C_{b}}{2\Delta PA^{2}}\right]V\;$$

where *t* is filtration time (s), *V* is permeate volume (L), and *C_b_* is foulant concentration (kg L^−1^). The mass of the deposited particles on the membrane surface (*M_d_*) was estimated based on a simple relationship between the accumulated volume of permeate (*V*, L) and foulant concentration (kg L^−1^), as shown in Equation (13).

(13) $$M_{d}=VC_{b}$$

## 3. Results and Discussion

### 3.1. Foulant Characteristics

**Figure 3:**
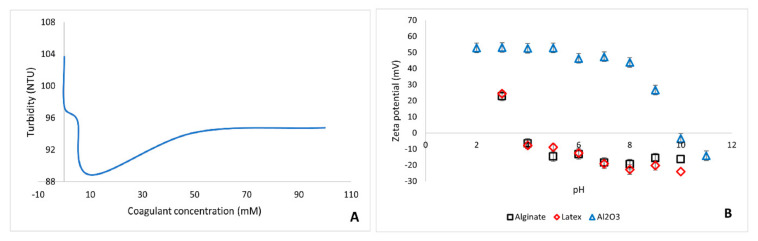
Graphs of critical coagulation concentration and zeta potential: (**A**)—Critical coagulation concentration (CCC) of 400 mg L^−1^ polystyrene carboxylated latex at pH 6.5 and (**B**)—zeta potential of alginate, latex, and Al_2_O_3_ as a function of pH.

Foulant size analysis revealed the following sizes for the foulants: 128 ± 2.7 nm for alginate, 167 ± 3.7 nm for latex, and 189 ± 4.1 nm for Al_2_O_3_. In the presence of calcium, latex had a particle size of 173 ± 2.9 nm, while the measurements of alginate size in the presence of calcium was cumbersome due to the aggregation nature of alginate. Figure 3A shows the critical coagulation concentration graph of polystyrene carboxylated latex with calcium chloride (used as coagulant). The turbidity of the latex suspension decreases with increase in calcium chloride concentration up until a calcium concentration of about 10 mM where the turbidity increased and leveled off. The critical coagulation concentration of 400 mg L^−1^ latex was reached at coagulant concentration of 990 mg L^−1^ calcium chloride (8.92 mM calcium chloride). Calcium concentration of 55 mgL^−1^ (0.5 mM calcium chloride), which was lower than the critical coagulation concentration, was used for all fouling experiments to ensure that no extra calcium ions neutralize the membrane surface, hence changing the membrane surface properties before fouling. It must be noted that the critical coagulation concentration is dependent on the foulant concentration.

Foulant zeta potential is important in predicting possible foulant–membrane interactions in terms of attractive and repulsive electrostatic interactions. The zeta potential of the foulants as a function of solution pH is presented in Figure 3B. Alginate and latex had similar trends of zeta potential at varying pH. At pH 7, which was the working pH for all fouling studies, alginate and latex had negative zeta potential, while Al_2_O_3_ had a positive zeta potential. The zeta potential of alginate and latex became more negative with an increase in solution pH due to the dissociation of the carboxylic functional groups at higher pH values. The zeta potential stabilized slightly at pH 7 to pH 10. Al_2_O_3_ particles had a zero point of charge at pH 9.8 and this was consistent with findings of other researchers who have reported the isoelectric point of Al_2_O_3_ to be between pH 8 and 9 [14]. The particles possessed a negative zeta potential above pH 10 due to the formation of oxide groups according to Equation (14) [14].

(14) $${\mathrm{Al}}_{2}O_{3}+\;2\mathrm{NaOH}+3H_{2}O\to 2{\mathrm{Na}}^{+}+\;2{\left[\mathrm{Al}{\left(\mathrm{OH}\right)}_{4}\right]}^{-}$$

### 3.2. Membrane Characteristics

**Figure 4:**
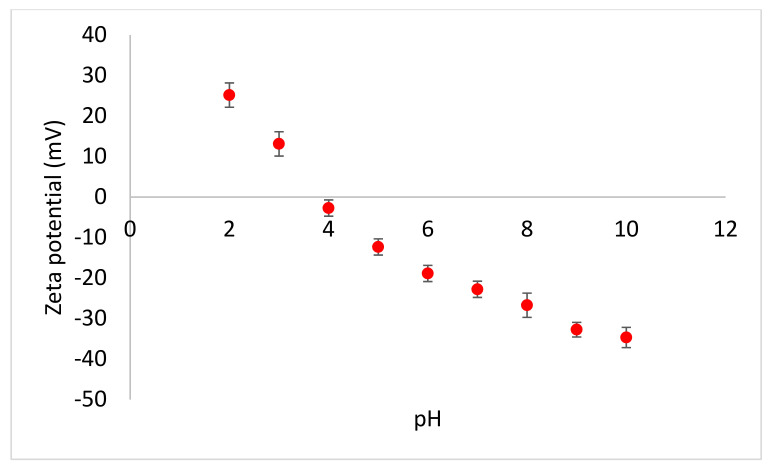
Zeta potential of the virgin NF-270 membrane as a function of pH.

The clean NF-270 membrane had a permeate flux (*J*_0_) of 60 L m^−2^ h^−1^ at applied pressure of 300 kPa, membrane resistance of 1.9 × 10^10^ m^−1^, and rejected (*R*_0_) 35% NaCl. The membrane was hydrophilic, with a water contact angle of 43°. At lower pH, the membrane had positive zeta potential, while negative zeta potential prevailed at pH values higher than pH 3.5 (Figure 4) due to the deprotonation of functional groups at elevated pH [26].

The measured zeta potential of the clean membrane concurred with literature results where the zeta potential of −8.0, −19.4, and −24.7 mV were reported at pH 4, 6, and, 8, respectively [27]. Generally, the zeta potential of the membrane became more negative with increasing pH. This could be attributed to the polyamide active layer which contains carboxylic and amine functional groups. The carboxylic functional groups dissociated at higher pH and the amine groups dissociated at very low pH to give positive zeta potential.

EDX spectra revealed that the NF-270 membrane was composed mainly of C, S, and O, and had a smooth surface with roughness of 4.1 nm [21].

### 3.3. Influence of Membrane Fouling on Permeate Flux

**Figure 5:**
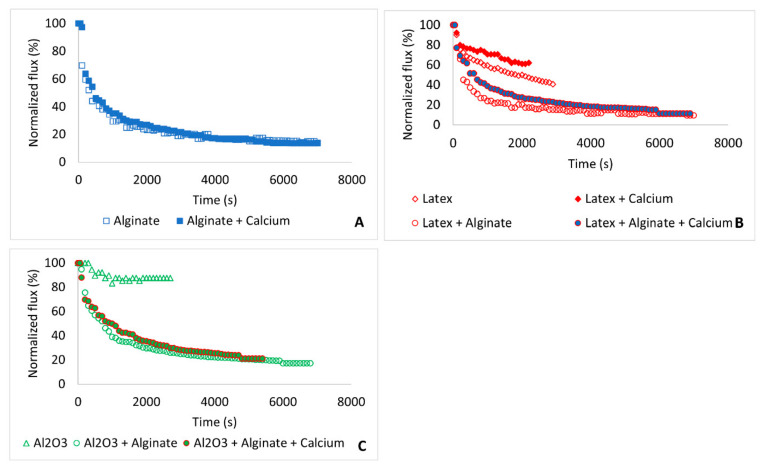
Flux decline profiles due to membrane fouling: (**A**)—Alginate fouling; (**B**)—latex fouling; and (**C**)—Al_2_O_3_ fouling.

The interplay between physical and chemical interactions governs membrane fouling [17,28]. Figure 5 shows the effect of fouling on permeate flux, where the normalized flux has been calculated from the flux of the fouled membrane (*J′*) divided by the initial flux (*J*_0_). Fouling by 266 mg L^−1^ alginate resulted in over 86% decline in permeate flux (Figure 5A). The addition of calcium ions did not influence the fouling trend as the flux decline profiles for alginate and alginate + calcium were more or less the same.

Fouling by 400 mg L^−1^ latex resulted in 59% decline in permeate flux, but the addition of calcium ions reduced the impact of latex fouling on flux decline and the flux declined by 35% (Figure 5B). Combined fouling by latex and alginate worsened membrane fouling and the flux decline curve mirrored that of fouling by alginate, showing more influence of alginate in combined fouling.

Fouling by Al_2_O_3_ (400 mg L^−1^) resulted in 13% flux decline (Figure 5C). However, combined fouling by Al_2_O_3_ and alginate worsened the permeate flux decline in the presence and absence of calcium ions. The fouling trend closely mirrored that of fouling by alginate alone, again showing the influence of alginate in membrane fouling. The aggravated flux decline in combined fouling was believed to be due to an increase in foulant concentration in the feed solution, as well as synergistic effects of membrane fouling by organic and colloidal foulants.

In a study by Zhu and Elimelech (1995), fouling by aluminum oxide in cross-flow mode did not result in flux decline, although fouling was favorable due to opposite charges of the foulant and membrane [29]. Membrane fouling is expected to be aggravated when the membrane and foulants bear opposite surface zeta potentials due to attractive membrane–foulant electrostatic interactions which promote foulant attachment on the membrane surface. It was believed that the formed monolayer of the foulant either did not provide enough cake resistance to permeate flow or was easily washed away at a cross-flow velocity of 0.2 ms^−1^. A similar observation was made in this study when fouling was performed in dead-end filtration mode; however, washing away of the fouling layer was irrelevant, implying that the cake layer did not exert enough hydraulic resistance to permeate flow.

Contrary to other studies which have noted more flux decline in the presence of calcium due to a decrease in surface zeta potential of the organic foulants and formation of cross-links between the organic foulants [4,9,30], this study noted lesser fouling (especially in the early stages of the fouling experiments) when calcium was added into the feed solution. This observation was attributed to formation of larger foulant aggregates with reduced resistance to permeate flow [1,31]. There was a rapid decline in flux within the first 300 s of fouling, but this was exceptional for fouling with Al_2_O_3_. This fouling behavior has been observed initially by other researchers who attributed it to pore restriction and deposition of the foulants on the membrane surface [32]. The gradual decline afterwards was due to compaction of the fouling layer [27]. The flux profiles presented in Figure 5 indicate that permeate flux in dead-end filtration may potentially become 0 Lm^−2^h^−1^ over time and this may depend on the foulant type and foulant concentration. Therefore, it may be necessary to recover the flux through backwashing where the flow is intermittently reversed to control fouling. During backwashing, the permeate water is used to flush the membrane and this decreases the net yield of treated water. Alternatively, flux recovery may be attempted through chemical cleaning using sodium hydroxide (NaOH), citric acid, ethylene diamine tetra-acetate (EDTA), chlorine (Cl_2_), and surfactants or detergents which have been shown to remove majority of the foulants including organic foulants, colloids, bio-foulants, and scalants [33].

In the next section, CECP effects and dominating resistance parameters influencing flux decline are investigated. These parameters were related to membrane properties as well as foulant properties.

### 3.4. Factors Governing Flux Decline and Their Interdependence

#### 3.4.1. Cake-Enhanced Concentration Polarization Effects

Cake-enhanced concentration polarization (CECP) has been cited as the major contributor to flux decline in fouling of NF membranes, especially in dead-end filtration. However, it is not clear whether the role of CECP is substantial for NF membranes that poorly reject salts. According to the solution–diffusion model, flux through a membrane increases linearly with applied pressure and solute permeability does not change in dense membranes [34]. However, solute permeability is independent of the applied pressure and solute concentration. Therefore, solute rejection reaches 100% at high fluxes based on Equation (15) [34,35].

(15) $$R_{0}=\left(\frac{J_{0}}{J_{0}+B}\right)\times 100$$

where *B* is the membrane salt permeability which was estimated at 3.1 × 10^−5^ g m^−2^.s from initial flux of 1.67 ms^−1^ (60 L m^−2^ h^−1^) and 35% salt rejection. Unlike solute permeability coefficient, solute rejection and salt flux depend on the applied pressure, where salt flux slowly increases with increasing flux. Similarly, solute rejection increases with flux due to the dilution effect of the increasing water flux. The solution diffusion model is widely used to predict the performance of nanofiltration and reverse osmosis membranes in water treatment as well as dialysis, gas separation and pervaporation [36,37,38,39].

**Figure 6:**
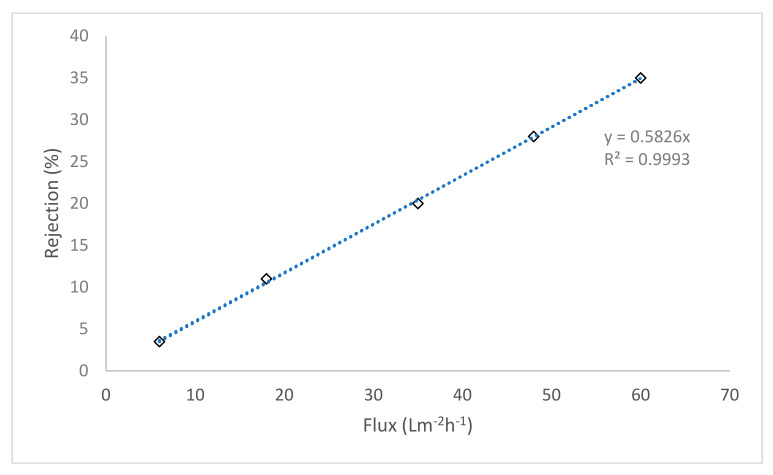
Salt rejection as a function of permeate flux for the virgin membrane.

Mahlangu et al. plotted rejection values as a function of flux for the virgin and fouled membranes and showed that the decline in rejection of salts was due to reduced water fluxes instead of CECP effects [9]. A similar approach was applied to investigate CECP effects for the dead-end fouling of NF-270 membrane that poorly rejects salts. The aim was to investigate if CECP effects are always substantial in membrane fouling or not. It was envisaged that CECP effects are not important when the background electrolyte salt is poorly rejected by the membrane, but fouling is controlled by other contributing factors such as calcium-NOM complexation, affinity interactions as well as cake filtration. Salt rejection (*R*_0_) was plotted as a function of permeate flux (*J*_0_) for the virgin membrane. Membrane permeate flux was adjusted by changing the applied pressure. It was noted that for the virgin membrane, salt rejection declined with a decrease in permeate flux (Figure 6).

Table 2 shows permeate flux and NaCl rejection of the fouled membranes after fouling at 300 kPa. Change in permeate flux (∆*J′*) and change in NaCl rejection (∆*R′*) was estimated from an initial flux (*J*_0_) of 60 L m^−2^ h^−1^ (1.67 × 10^−5^ ms^−1^) and initial salt rejection (*R*_0_) of 35%, respectively, as follows: ∆*J′* was calculated from (1 − (*J′*/*J*_0_) × 100) and ∆*R′* was calculated from (1 − *R′*/*R*_0_) × 100), where *R*_0_ and *J*_0_ are the rejection and flux before fouling, respectively, while *R′* and *J′* are the rejection and flux of the fouled membranes, respectively.

From Figure 6, rejection changes linearly with permeate flux (R^2^ = 0.9998) and the ratio of change in rejection over time against change in flux over time (∆*R*_0_/∆*J*_0_) ≈ 1. Therefore, for fouled membranes, a ratio of ∆*R′*/∆*J′* greater than 1 implies substantial CECP effects, as the rejection declined more than the flux, while a ∆*R′*/∆*J′* less than 1 shows that a substantial decline in flux did not result in more decline in salt rejection; therefore, the fouled membrane rejected more NaCl compared to the virgin membrane at a similar flux. Except for fouling with latex in the presence of calcium, CECP effects did not seem to play a major role in fouling of the low salt rejecting membrane (Table 2). It appears that CECP effects are not important in fouling of membranes that poorly reject salts. Based on the minor influence of CECP effects on flux decline, hydraulic resistances of the different type of foulants (single and mixed) were investigated as major contributors to flux decline in fouling of the NF-270 membrane.

#### 3.4.2. Resistance Parameters

Table 3 shows different resistance parameters resulting from organic, colloidal, and combined fouling in the presence and absence of calcium. The specific resistance (α) for the individual foulants was always higher than specific resistances of the same foulant in the presence of calcium ions. This observation also applied for combined fouling experiments. This reduction in α explains the reduced fouling rate (especially in the early stages of fouling) when fouling was conducted with the addition of calcium. For example, the flux decline was less when fouling was conducted with alginate + calcium compared to fouling with only alginate after 2000 s of fouling (Figure 5A) and flux decline for latex + calcium was lower than latex fouling (Figure 5B).

**Figure 7:**
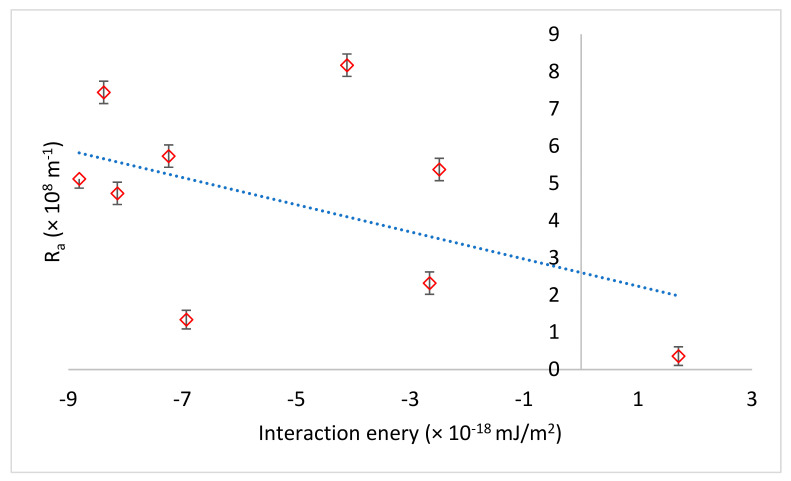
Resistance due to foulant adsorption on the membrane surface as a function of membrane–foulant interaction energy.

Resistance due to the adsorption of foulants on the membrane surface (*R_a_*) followed a similar pattern as specific resistance, where the addition of calcium decreased *R_a_*. This was contrary to expectations where more foulant adsorption on the membrane surface was expected due to reduction in membrane–foulant repulsive interactions in the presence of calcium. However, there was not only electrostatic interaction control fouling, but also non-electrostatic membrane–foulant interaction (affinity interactions). The presence of calcium ions reduced the foulants’ affinity for the membrane surface. When Ra was plotted as a function of membrane–foulant non-electrostatic interaction energy (Figure 7), it was noted that Ra decreased as membrane–foulant non-electrostatic interactions became repulsive (i.e., ∆*G_i_* became less negative). Non-electrostatic interactions account for van der Waals interactions, H-bonding, dielectric effects, and hydrophobic attractions [34].

There was no conclusive observable trend on hydraulic resistance due to pore blocking (*R_p_*). This was expected as pore blocking by the foulants which were way larger than the membrane pore size (average pore diameter of 0.84 nm) was not possible. Therefore, pore blocking may not be substantial in fouling of nanofiltration membranes, more especially for experiments conducted in this study. Resistance due to cake filtration (*R_c_*) had a greater contribution in flux decline for all foulants. For alginate fouling and combined Al_2_O_3_ + alginate fouling experiments, the addition of calcium increased *R_c_*, indicating the formation of dense fouling layers due to calcium bridging on the carboxylic functional groups of sodium alginate. Though the presence of calcium improved *R_c_* for alginate and combined Al_2_O_3_ + alginate fouling, there was no noticeable difference in the flux profiles for alginate and combined Al_2_O_3_ + alginate fouling in the presence of calcium. This can be attributed to the noticeable decrease in specific resistance in the presence calcium (Table 3).

Regarding latex fouling, the addition of calcium decreased *R_c_* due to the formation of larger latex particles (173 ± 2.9 nm) which resulted in a fouling layer with larger pores and higher porosity according to the Carmen–Kozeny Equation [31]. Decrease in *R_c_* was also noted for combined latex + alginate in the presence of calcium and this may be attributed to competition for calcium ions between the carboxylic functional groups of alginate and latex. This competition may also disturb uniform formation of alginate–calcium bridges.

**Figure 8:**
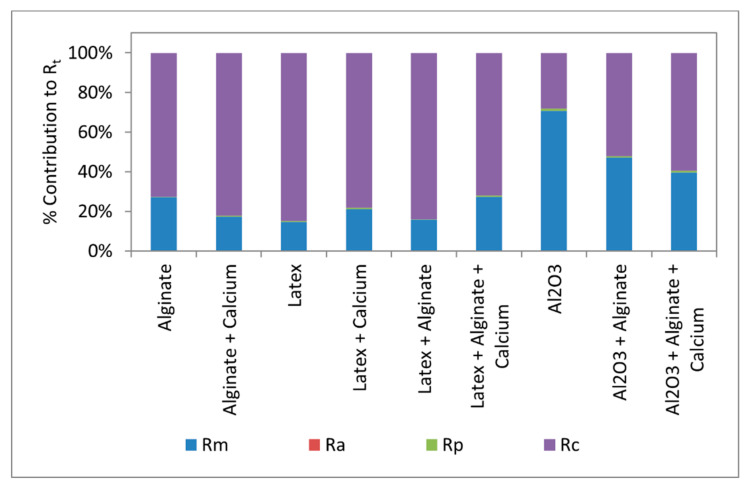
Contribution of *R_m_*, *R_a_*, *R_p_*, and *R_c_* to the total resistance.

The contribution of the individual resistance parameters to the total resistance are presented in Figure 8. Generally, the contribution of *R_p_* and *R_a_* to the total resistance or *R_t_* was minimal. This observation is in agreement with previous results reported by Listiarini and co-workers [1]. Except for fouling with Al_2_O_3_, *R_c_* contributed more to the total resistance leading to flux decline. The presence of Al_2_O_3_ in combined Al_2_O_3_ + fouling resulted in lower *R_c_* compared to that of fouling by alginate alone. This observation is consistent with findings of Listiarini and co-workers who conducted fouling of nanofiltration membranes in the dead-end filtration mode where the presence of alum in combined alginate + alum fouling reduced *R_c_* [1]. Again, the presence of Al_2_O_3_ might have disturbed the formation of the uniform alginate cake layer on the membrane surface.

Table 4 shows the mass of the deposited foulants (*M_d_*), the pure water permeability (*L_p_*), and the fouling index (*FI*) of the clean and fouled membranes. The pure water permeability of the fouled membranes was calculated by dividing the permeate flux by the applied pressure (300 kPa). From the table, it is apparent that the membrane pure water permeability decreased due to fouling. It can further be observed from the table that Al_2_O_3_ had the least influence in membrane fouling, even though the mass of Al_2_O_3_ deposited on the membrane surface was more than that of alginate. On the other hand, alginate, latex, and their combination resulted in more than 70% decline in membrane pure water permeability. Nghiem and co-workers noted an increase in flux decline with increasing foulant mass deposited on the membrane surface [27]. The authors made a comparison with reference to one foulant type (humic acid). In this study, the extent of fouling could not be related to the mass of the deposited foulant deposited on the membrane surface due to differences in fouling behaviors and physico-chemical properties between the foulants utilized. However, additional experiments (results not shown) showed lower flux decline at lower foulant concentration for all foulants and this was consistent with findings from the literature [27].

### 3.5. Verification of Foulant Layer Composition

Foulant deposition on the membrane surface was confirmed using scanning electron microscopy (SEM) imaging while the cake layer composition was verified using EDX and Fourier transform infrared (FTIR) spectroscopy.

**Figure 9:**
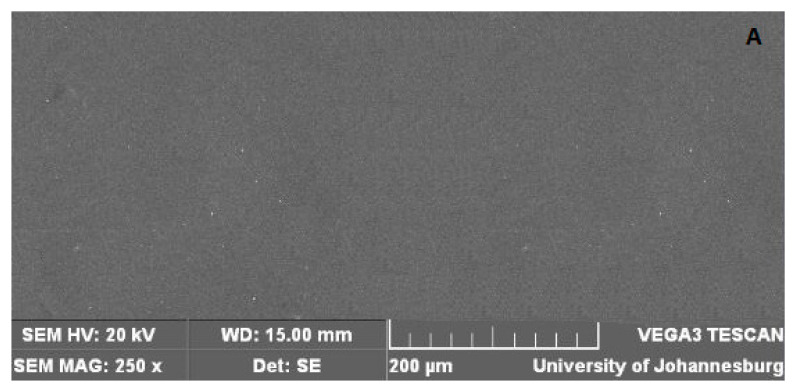

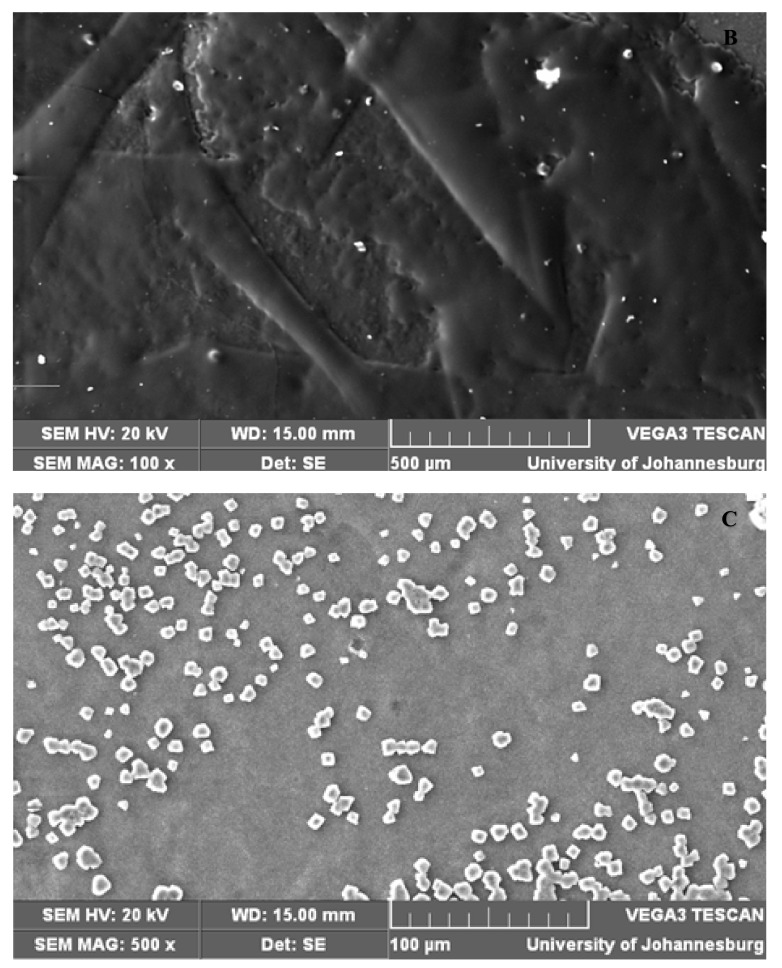
SEM micrographs of the virgin and fouled NF-270 membranes: (**A**)—Virgin membrane; (**B**)—latex + calcium-fouled membrane; (**C**)—Al_2_O_3_-fouled membrane.

Figure 9 shows SEM micrographs of the virgin membrane (Figure 9A) and fouled NF-270 membrane (Figure 9B,C), while EDX results of the membranes are presented in the Supplementary Materials. From all the EDX micrographs, a sodium (Na) peak was observed (see Figure S1 in Supplementary Materials). This peak originated from NaCl used as the background electrolyte. Sodium was also one of the constituents of alginate which was used in fouling experiments. Fouling by alginate resulted in the formation of a cake layer that completely covered the membrane surface (Figure S2A in Supplementary Materials). In the presence of calcium, dense fouling layers (completely covering the membrane surface) were formed (Figure S2B in Supplementary Materials). This suppressed sulfur (S) peaks but resulted in the detection of new calcium (Ca) peaks. From Figure S2C in the Supplementary Materials, it is apparent that fouling by latex did not result in a denser fouling layer compared to latex fouling in the presence of alginate and calcium (Figure 9B). Al_2_O_3_ fouling was clearly visible from SEM images and this was further confirmed by EDX analysis (Figure 9C). However, Al_2_O_3_ particles were sparsely distributed on the membrane surface. Upon addition of alginate and calcium ions, a dense fouling layer was noted for combined Al_2_O_3_ fouling, and aluminum could still be detected using EDX (Figure S2D in Supplementary Materials). Combined fouling appeared to result in more fouling on the membrane surface compared to individual fouling as evidenced by SEM images of the fouled membranes.

**Figure 10:**
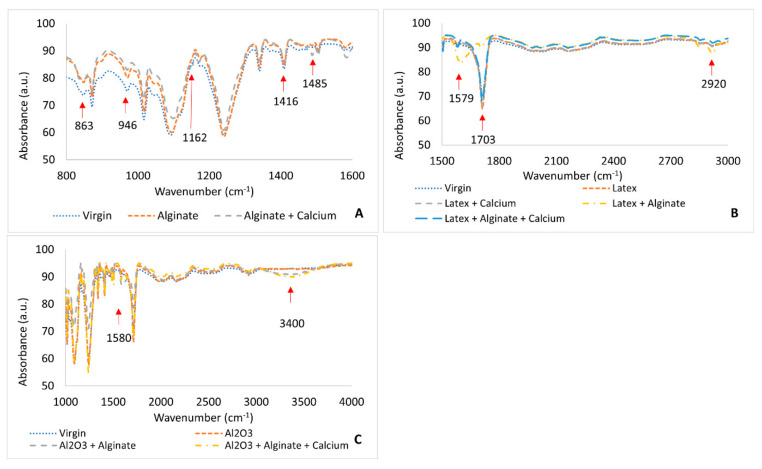
FTIR spectra of virgin and fouled NF-270 membrane coupons; (**A**)—Alginate-fouled membrane; (**B**)—latex and combined latex-fouled membranes; (**C**)—Al_2_O_3_ and combined Al_2_O_3_-fouled membrane.

Figure 10 shows FTIR spectra of the virgin and fouled membrane coupons. The spectra were used to further confirm the deposition and composition of the fouling layer on the membrane surface in addition to SEM/EDX images.

From the alginate-fouled membranes (Figure 10A), noticeable adsorption bands were observed at 863, 946, 1162, 1416, and 1485 cm^−1^ due to the deformation vibration of β-C_1_-H groups (863 cm^−1^), C-O stretching (946 cm^−1^), carboxyl anions asymmetric and symmetric vibrations (1416 cm^−1^) [40,41], C-O-C and O-H stretching vibrations (1162 cm^−1^) [42], and Na vibration bands (1485 cm^−1^) [43]. For the membrane fouled with latex (Figure 10B), there were distinguishable bands at 1579, 1703, and 2920 cm^−1^. The 1579 cm^−1^ was due to C-O-O functional groups and the 2920 cm^−1^ band was characteristic of latex [44] . The band at 1703 cm^−1^ was distorted on fouling with combined latex + alginate. This band was previously determined in FTIR studies of latex [45]. When the membrane was fouled with Al_2_O_3_, there was an absorption band at 3400 and 1580 cm^−1^, which was attributed to OH groups bonded via hydrogen bridges and symmetric and asymmetric C-O stretching vibrations (Figure 10C). The 3400 cm^−1^ band was noticed only on combined fouling of Al_2_O_3_ + alginate [46].

## 4. Conclusions

This study showed the effect of fouling on membrane performance in terms of permeate flux. Sodium alginate had more impact on fouling, as revealed by the flux declining more when fouling was conducted with alginate and combined alginate + colloids. The addition of calcium ions did not seem to worsen flux decline, as reported by other researchers. The fouling order was alginate > latex > Al_2_O_3_, where Al_2_O_3_ did not seem to have an impact on flux decline. Cake-enhanced concentration polarization effects did not play a role in flux decline for the membrane that poorly rejected salts. Addition of calcium ions decreased specific resistance of the fouling layers. Organic and colloidal foulants did adsorb on the membrane surface as membrane–foulant non-electrostatic interactions were attractive (∆*G_i_* was negative), and resistance due to foulant adsorbing on the membrane surface increased with foulant affinity for the membrane surface. Membrane pore blocking was not important for the tight membrane, and the major flux decline was due to cake resistance, where the fouling layer exerted more resistance to water flow through the membrane. SEM images revealed dense fouling layers for combined fouling due to increased foulant concentration, and the foulants completely covered the membrane surface. EDX and FTIR confirmed through functional groups that the foulants were indeed deposited on the membrane surface.

## Figures and Tables

**Table 1:** Foulant concentrations used in fouling studies. The background electrolyte concentration was maintained at 10 mM with the addition of NaCl.

Membrane Type	Foulant Concentration (mg L^−1^)			Calcium (mg L^−1^)
	Alginate	Latex	Al_2_O_3_	
Alginate	266	0.0	0.0	0.0
Alginate + Calcium	266	0.0	0.0	55.5
Latex	0.0	400	0.0	0.0
Latex + Calcium	0.0	400	0.0	55.5
Latex + Alginate	266	400	0.0	0.0
Latex + Alginate + Calcium	266	400	0.0	55.5
Al_2_O_3_	0.0	0.0	400	0.0
Al_2_O_3_ + Alginate	266	0.0	400	0.0
Al_2_O_3_ + Alginate + Calcium	266	0.0	400	55.5

**Table 2:** Permeate flux (*J′*), salt rejection (*R′*), and ratio of change in rejection against change in permeate flux (∆*R′*/∆*J*′).

	*J′* (Lm^−2^ h^−1^)	∆*J′* (%)	*R′* (%)	∆*R′* (%)	∆*R′*/∆*J′*
Alginate	8.4 ± 0.4	86 ± 0.3	1.1 ± 0.1	97 ± 0.2	1.1 ± 0.2
Alginate + Calcium	7.8 ± 0.2	87 ± 0.2	1.1 ± 0.1	97 ± 0.1	1.1 ± 0.1
Latex	24.6 ± 0.3	59 ± 0.2	14.4 ± 0.2	59 ± 0.2	1.0 ± 0.2
Latex + Calcium	31.0 ± 0.2	48 ± 0.3	21.7 ± 0.3	38 ± 0.2	0.8 ± 0.2
Latex + Alginate	5.5 ± 0.2	91 ± 0.3	3.2 ± 0.2	91 ± 0.2	1.0 ± 0.2
Latex + Alginate + Calcium	6.6 ± 0.3	89 ± 0.2	3.9 ± 0.1	89 ± 0.2	1.0 ± 0.2
Al_2_O_3_	52.2 ± 0.4	13 ± 0.3	30.5 ± 0.3	13 ± 0.3	1.0 ± 0.3
Al_2_O_3_ + Alginate	10.2 ± 0.3	83 ± 0.2	6.0 ± 0.3	82 ± 0.3	1.0 ± 0.3
Al_2_O_3_ + Alginate + Calcium	12.6 ± 0.4	79 ± 0.3	7.4 ± 0.3	79 ± 0.3	1.0 ± 0.3

**Table 3:** Resistances due to fouling in the presence and absence of calcium ions.

	α × 10^10^ (mk g^−1^)	*R_a_* × 10^8^ (m^−1^)	*R_p_* × 10^8^ (m^−1^)	*R_c_* × 10^10^ (m^−1^)
Alginate	4.07 ± 0.3	8.17 ± 0.2	1.59 ± 0.2	4.80 ± 0.2
Alginate + Calcium	3.55 ± 0.2	4.73 ± 0.4	5.76 ± 0.4	8.51 ± 0.4
Latex	3.32 ± 0.2	2.32 ± 0.2	5.81 ± 0.4	10.4 ± 0.5
Latex + Calcium	3.25 ± 0.3	1.34 ± 0.1	7.07 ± 0.3	6.62 ± 0.3
Latex + Alginate	1.84 ± 0.1	7.44 ± 0.4	2.71 ± 0.2	9.48 ± 0.3
Latex + Alginate + Calcium	1.81 ± 0.1	5.37 ± 0.3	3.78 ± 0.3	4.71 ± 0.4
Al_2_O_3_	3.02 ± 0.3	0.36 ± 0.1	2.68 ± 0.2	0.71 ± 0.1
Al_2_O_3_ + Alginate	1.44 ± 0.2	5.73 ± 0.4	3.05 ± 0.3	1.98 ± 0.3
Al_2_O_3_ + Alginate + Calcium	1.02 ± 0.1	5.12 ± 0.3	4.39 ± 0.3	2.68 ± 0.3

**Table 4:** Cake parameters and resultant membrane performance in terms of pure water permeability (*L_p_*).

	*M_d_* (mg)	*L_p_* (Lm^−2^ h^−1^ kPa^−1^)	*FI* (%)
Clean	0.0	0.205 ± 0.01	0.0
Alginate	53.2	0.028 ± .001	86 ± 0.2
Alginate + Calcium	53.2	0.027 ± 0.01	87 ± 0.3
Latex	80.0	0.084 ± 0.01	59 ± 0.2
Latex + Calcium	80.0	0.127 ± 0.01	38 ± 0.4
Latex + Alginate	133.2	0.018 ± 0.01	91 ± 0.6
Latex + Alginate + Calcium	133.2	0.023 ± 0.01	89 ± 0.5
Al_2_O_3_	80.0	0.179 ± 0.01	13 ± 0.2
Al_2_O_3_ + Alginate	133.2	0.035 ± 0.01	83 ± 0.6
Al_2_O_3_ + Alginate + Calcium	133.2	0.043 ± 0.01	79 ± 0.7

## Supplementary Materials

The following are available online at https://www.mdpi.com/2077-0375/11/1/47/s1, Figure S1. EDS micrographs of the virgin and fouled NF-270 membranes: (A)—Virgin membrane; (B)—latex + calcium-fouled membrane; (C)—Al_2_O_3_-fouled membrane; Figure S2: SEM/EDS micrographs of fouled NF-270 membranes: A—alginate fouling; B—alginate + calcium; C—latex; D—Al_2_O_3_ + alginate + calcium.
