# Supplementary material for: Interdependence of Contributing Factors Governing Dead-End Fouling of Nanofiltration Membranes
Source: Membranes (Basel). 2021 Jan 12;11(1):47. doi: 10.3390/membranes11010047 (PMC7827496; doi:10.3390/membranes11010047)
Supplement: Supplementary file 1 [file membranes-11-00047-s001.pdf]

membranes

MDPI

Article

Interdependence of Contributing Factors Governing Dead-End Fouling of Nanofiltration Membranes

Oranso Themba Mahlangu and Bhekie Brilliance Mamba*

Institute for Nanotechnology and Water Sustainability, College of Engineering, Science and Technology, University of South Africa, Florida Science Campus, 1709 Roodepoort, South Africa; orathem@gmail.com (O.T.M)
* Correspondence: Mambabb@unisa.ac.za

Received: date; Accepted: date; Published: date

Supplementary information

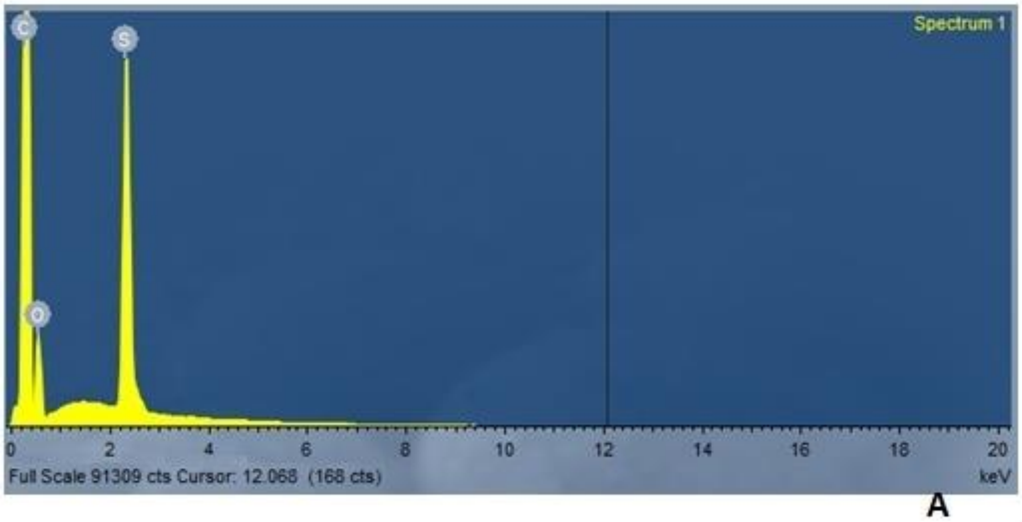

A

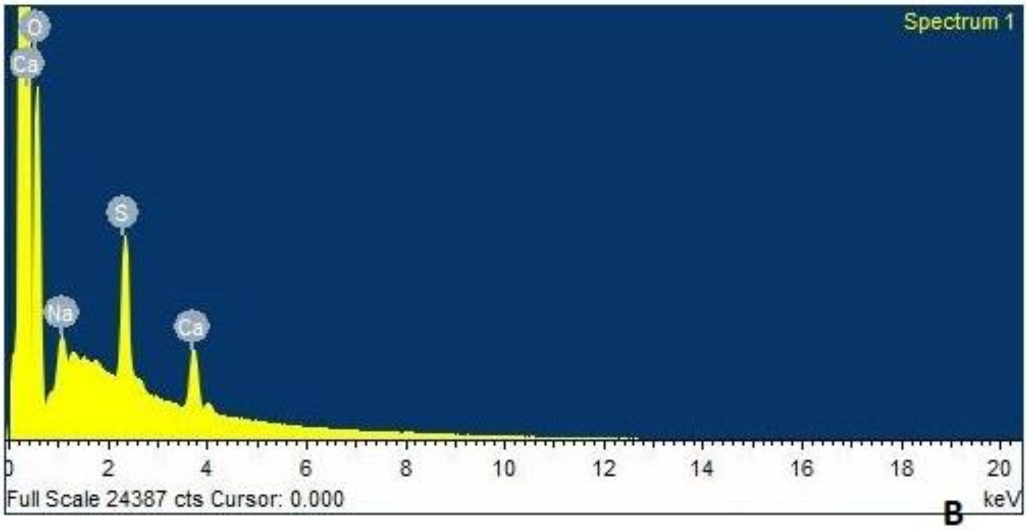

B

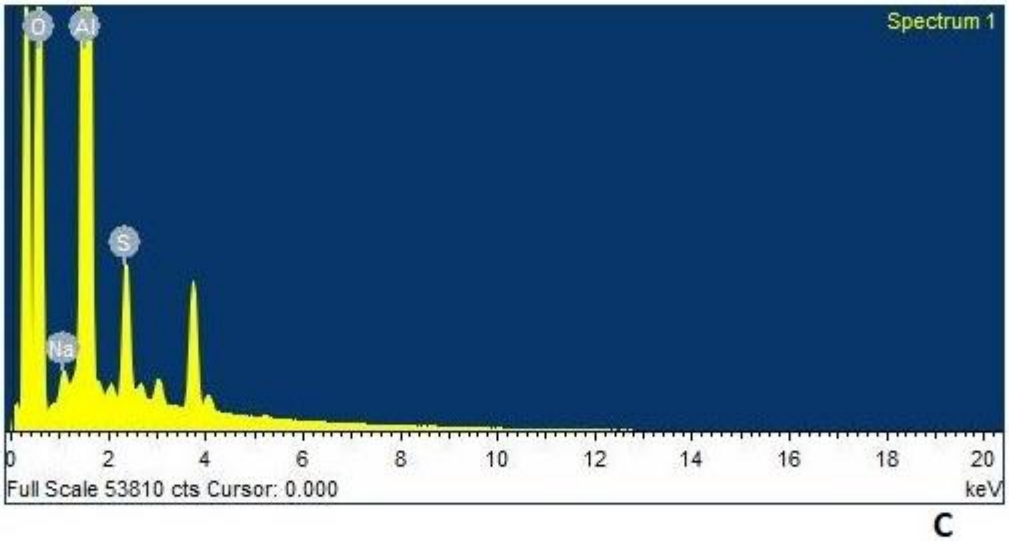

Figure S1. EDS micrographs of the virgin and fouled NF-270 membranes: (**A**)—Virgin membrane; (**B**)—latex + calcium-fouled membrane; (**C**)—Al_2O_3-fouled membrane.

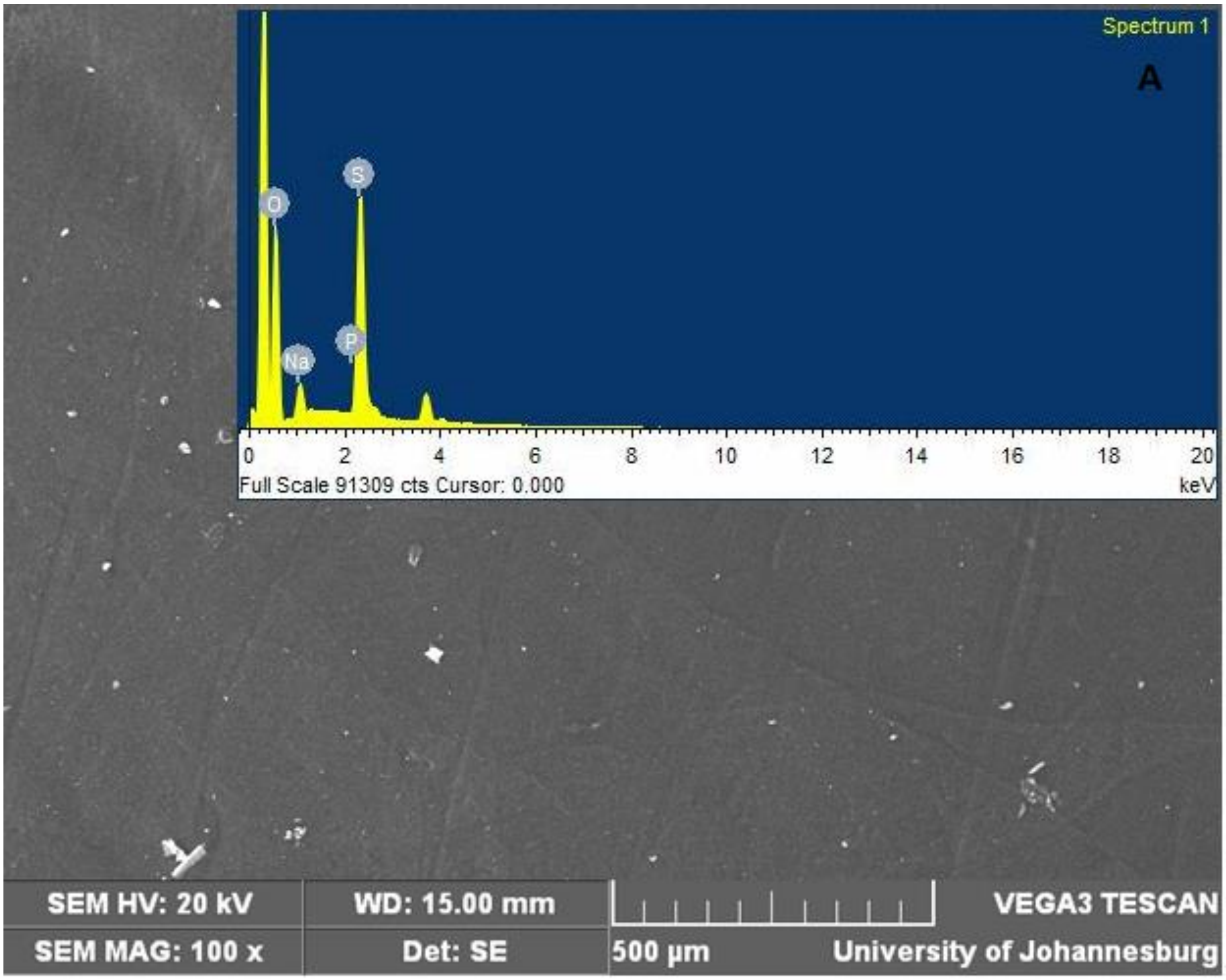

membranes

MDPI

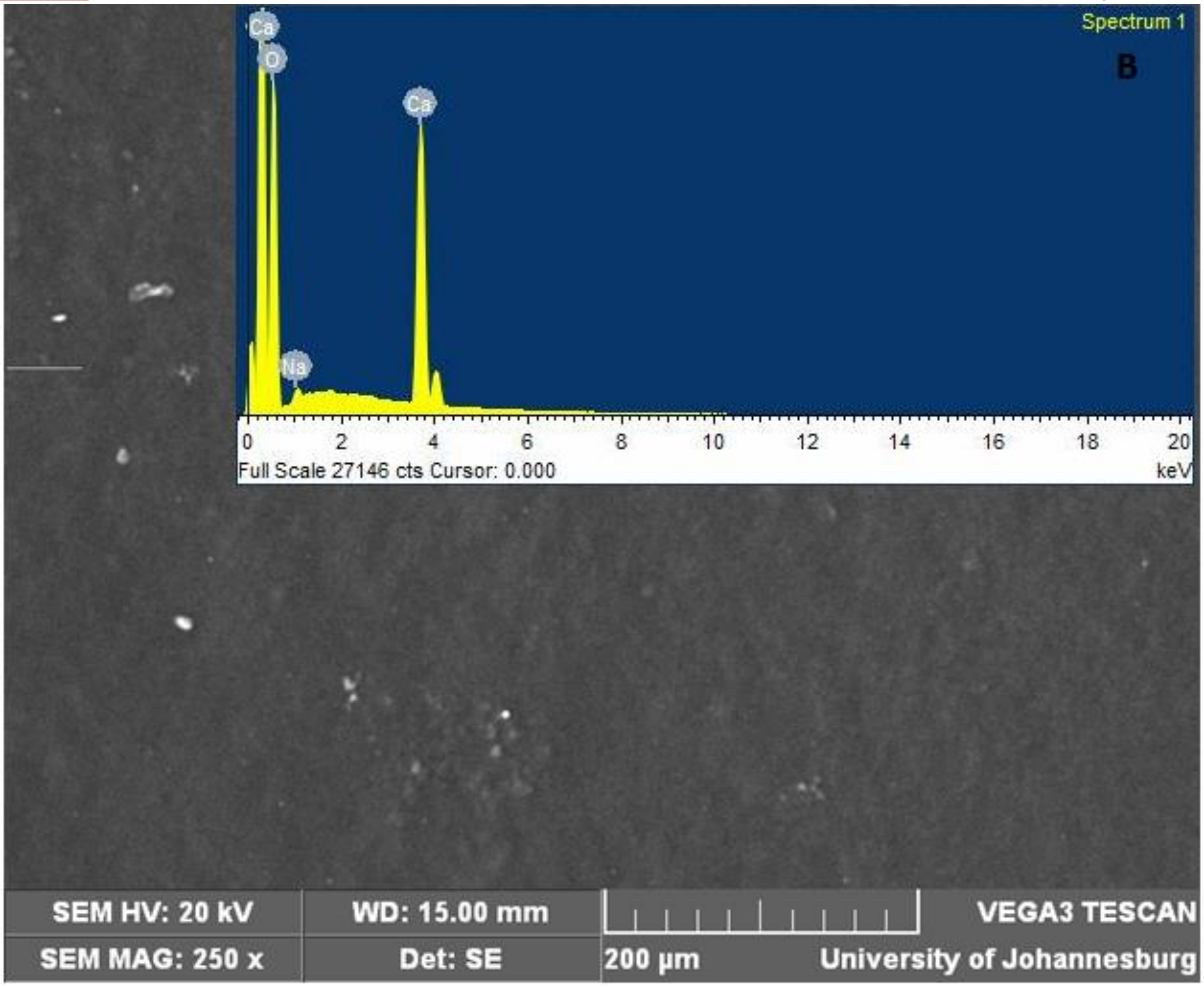

membranes

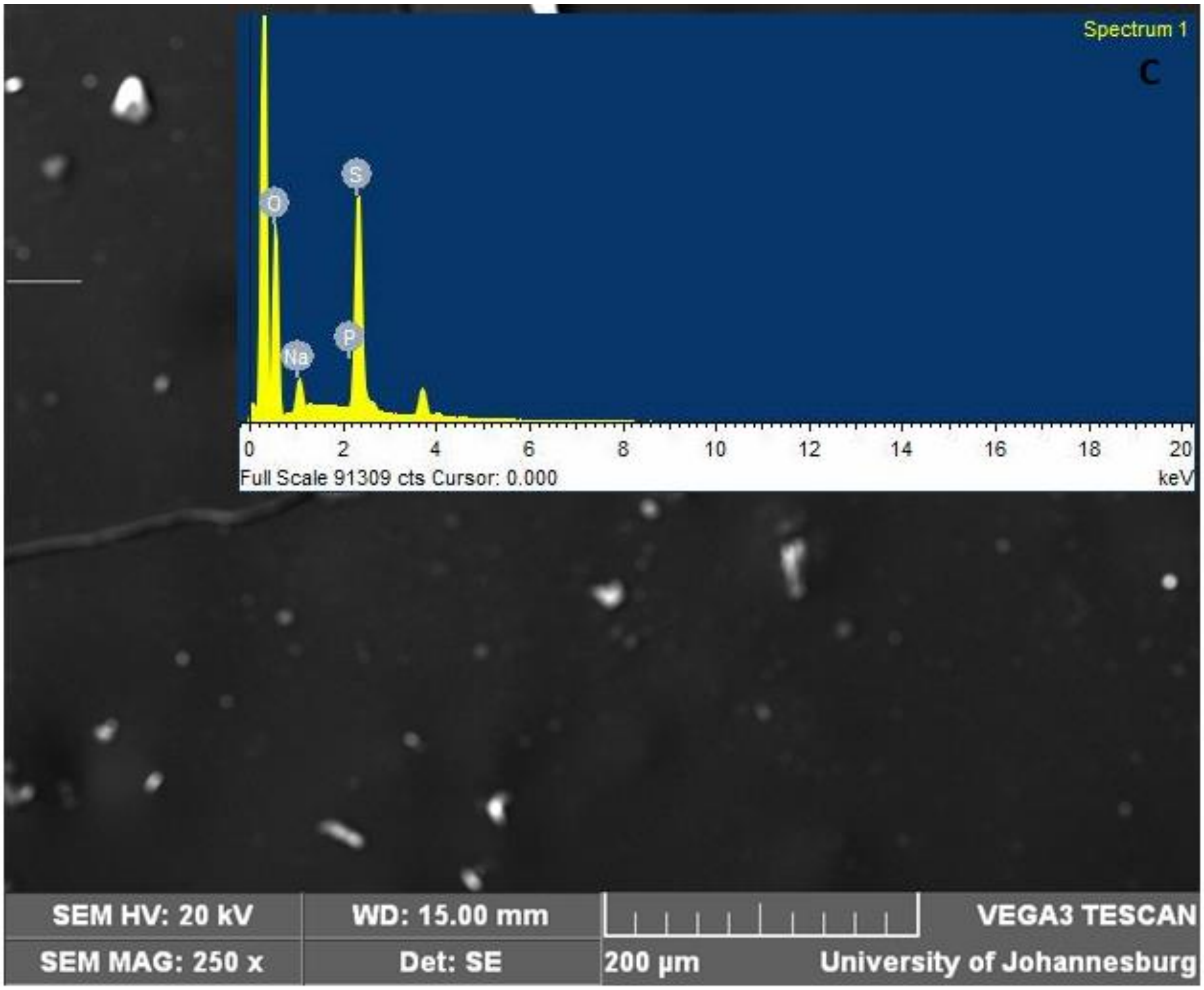
Spectrum 1
C
O
S
Na
P
0 2 4 6 8 10 12 14 16 18 20
Full Scale 91309 cts Cursor: 0.000
keV
SEM HV: 20 kV
WD: 15.00 mm
VEGA3 TESCAN
SEM MAG: 250 x
Det: SE
200 µm
University of Johannesburg

membranes

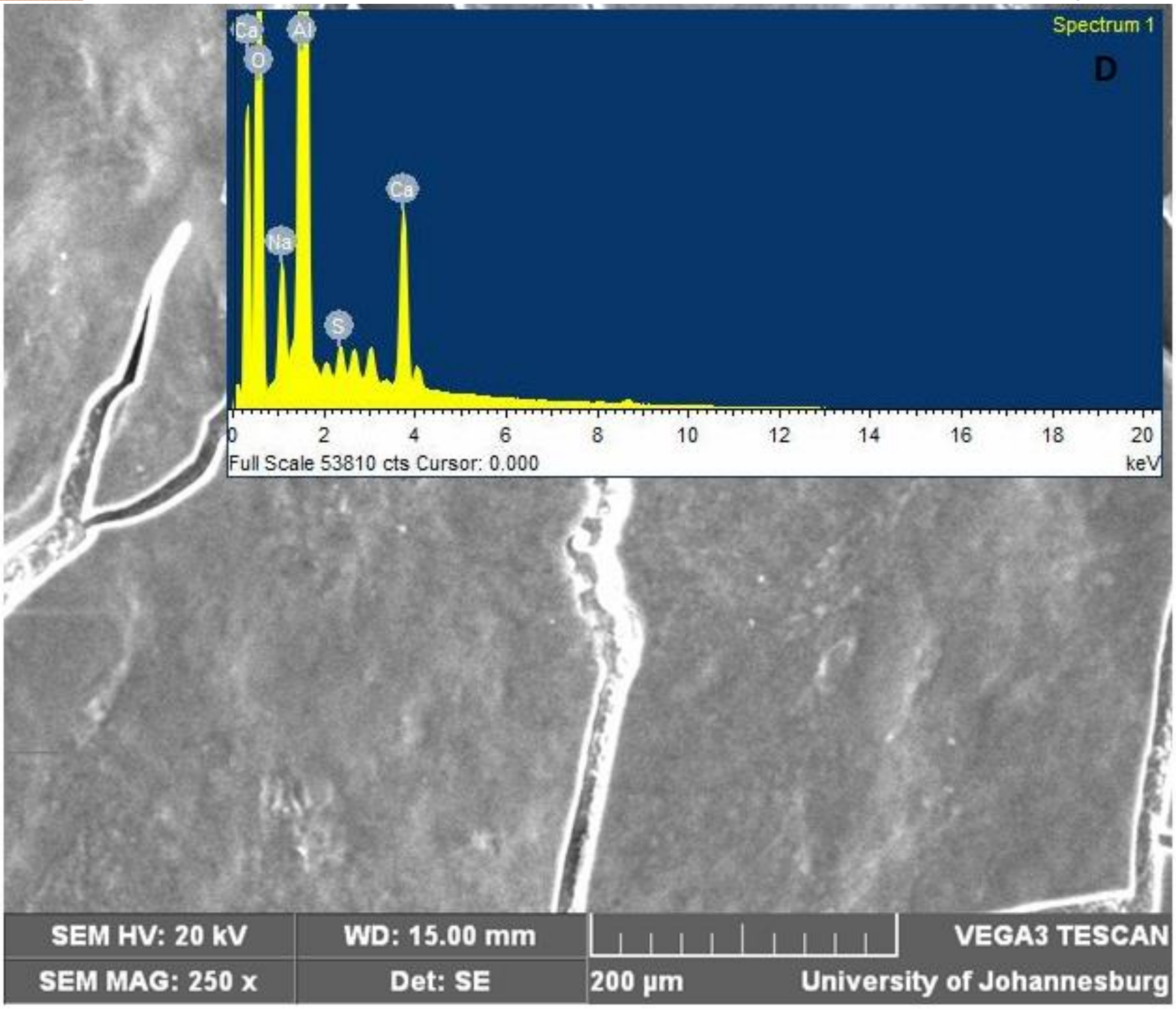

Figure S2. SEM/EDS micrographs of fouled NF-270 membranes: A – alginate fouling; B – alginate + Calcium; C – latex; D – Al2O3 + alginate + Calcium
